# Patented Drug Extension Strategies on Healthcare Spending: A Cost-Evaluation Analysis

**DOI:** 10.1371/journal.pmed.1001460

**Published:** 2013-06-04

**Authors:** Nathalie Vernaz, Guy Haller, François Girardin, Benedikt Huttner, Christophe Combescure, Pierre Dayer, Daniel Muscionico, Jean-Luc Salomon, Pascal Bonnabry

**Affiliations:** 1 Pharmacy, Geneva University Hospitals, Geneva, Switzerland; 2 School of Pharmaceutical Sciences, University of Geneva, University of Lausanne, Geneva, Switzerland; 3 Department of Anesthesiology, Pharmacology, Intensive Care, Geneva University Hospitals, Geneva, Switzerland; 4 CRC and Division of Clinical Epidemiology, Department of Health and Community Medicine, University of Geneva, Geneva, Switzerland; 5 Department of Epidemiology and Preventive Medicine, Health Services Management and Research Unit, Monash University, Melbourne, Australia; 6 Clinical Psychopharmacology Unit, Service of Clinical Pharmacy and Toxicology, Geneva University Hospitals, Geneva, Switzerland; 7 Medical Directorate, Geneva University Hospitals, Geneva, Switzerland; 8 Centre for Health Economics, University of York, York, United Kingdom; 9 Infection Control Program, Geneva University Hospitals, Geneva, Switzerland; 10 Invoice Office, OFAC, Geneva, Switzerland; Brigham and Womens Hospital, United States of America

## Abstract

**Background:**

Drug manufacturers have developed “evergreening” strategies to compete with generic medication after patent termination. These include marketing of slightly modified follow-on drugs. We aimed to estimate the financial impact of these drugs on overall healthcare costs and also to examine the impact of listing these drugs in hospital restrictive drug formularies (RDFs) on the healthcare system as a whole (“spillover effect”).

**Methods and Findings:**

We used hospital and community pharmacy invoice office data in the Swiss canton of Geneva to calculate utilisation of eight follow-on drugs in defined daily doses between 2000 and 2008. “Extra costs” were calculated for three different scenarios assuming replacement with the corresponding generic equivalent for prescriptions of (1) all brand (i.e., initially patented) drugs, (2) all follow-on drugs, or (3) brand and follow-on drugs. To examine the financial spillover effect we calculated a monthly follow-on drug market share in defined daily doses for medications prescribed by hospital physicians but dispensed in community pharmacies, in comparison to drugs prescribed by non-hospital physicians in the community.

Estimated “extra costs” over the study period were €15.9 (95% CI 15.5; 16.2) million for scenario 1, €14.4 (95% CI 14.1; 14.7) million for scenario 2, and €30.3 (95% CI 29.8; 30.8) million for scenario 3. The impact of strictly switching all patients using proton-pump inhibitors to esomeprazole at admission resulted in a spillover “extra cost” of €330,300 (95% CI 276,100; 383,800), whereas strictly switching to generic cetirizine resulted in savings of €7,700 (95% CI 4,100; 11,100). Overall we estimated that the RDF resulted in “extra costs” of €503,600 (95% CI 444,500; 563,100).

**Conclusions:**

Evergreening strategies have been successful in maintaining market share in Geneva, offsetting competition by generics and cost containment policies. Hospitals may be contributing to increased overall healthcare costs by listing follow-on drugs in their RDF. Therefore, healthcare providers and policy makers should be aware of the impact of evergreening strategies.

*Please see later in the article for the Editors' Summary*

## Abstract 2

In a cost-evaluation analysis of pharmacy invoice data in one Canton in Switzerland, Nathalie Vernaz and colleagues find that “evergreening” strategies pursued by drug manufacturers have been successful in maintaining market share and contribute to increased overall healthcare costs.

*Please see later in the article for the Editors' Summary*

## Editors' Summary

**Background:**

The development of a new medical drug—from discovery of a new compound to regulatory approval for its use—can take many years and cost millions of dollars. In 1995 the World Trade Organization adopted an international law (Trade-Related Aspects of Intellectual Property Rights—TRIPS) by which pharmaceutical companies can protect their intellectual property through patents. Under TRIPS, pharmaceutical companies are granted exclusive manufacturing rights for up to 20 years for each new drug, generating large revenues that often exceed initial investments costs, thus providing an incentive for pharmaceutical companies to continue to invest in the research and development of new drugs. However, recent stricter regulatory procedures for drug approval, national price control policies, and increased competition from generic manufacturers (that produce drugs similar to the brand drug once the patent has expired) have meant that pharmaceutical company profits have increasingly come under pressure.

**Why Was This Study Done?:**

One of the tactics that pharmaceutical companies currently use in response to this situation is to extend their market monopoly. This practice is known as “evergreening” and refers to the situation in which pharmaceutical companies slightly change the formulation of their brand drug into “follow on” drugs, for example, by combining formulations or producing slow-release forms, so that they can extend the patent. The impact of such follow-on drugs on overall healthcare costs in high-resource settings is unclear and has received little attention. In this study, the researchers assessed the overall costs associated with the prescribing of follow-on drugs in the Swiss canton of Geneva.

**What Did the Researchers Do and Find?:**

The researchers identified prescriptions of eight follow-on drugs issued by hospital and community pharmacists in Geneva between 2000 and 2008. To analyze the impact of evergreening strategies on healthcare spending, they calculated the market share score (an indicator of market competitiveness) for all prescriptions of the originally patented (brand) drug, the follow-on drug, and generic versions of the drug. The researchers then used hospital and community databases to analyze the costs of replacing brand and/or follow-on drugs with a corresponding generic drug (when available) under three scenarios (1) replacing all brand drug prescriptions, (2) replacing all follow-on drug prescriptions, and (3) replacing both follow-on and brand prescriptions.

Using these methods, the researchers found that over the study period, the number of patients receiving either a brand or follow-on drug increased from 56,686 patients in 2001 to 131,193 patients in 2008. The total cost for all studied drugs was €171.5 million, of which €103.2 million was for brand drugs, €41.1 million was for follow-on drugs, and €27.2 million was for generic drugs. Based on scenario 1 (all brand drugs being replaced by generics) and scenario 2 (all follow-on drugs being replaced by generics), over the study period, the healthcare system could have saved €15.9 million and €14.4 million in extra costs, respectively. The researchers also found some evidence that hospital prescribing patterns (through a restrictive drug formulary) influenced prescribing in the community: over the study period, the influence of hospital prescription patterns on the community resulted in an extra cost of €503,600 (mainly attributable to two drugs, esomeprazole and escitalopram). However, this influence also resulted in some savings because of a generic drug listed in the hospital formulary: use of the generic version of the drug cetirizine resulted in savings of €7,700.

**What Do These Findings Mean?:**

These findings show that in a high-income setting, evergreening strategies developed by pharmaceutical companies for follow-on drugs substantially contributed to an increase in overall healthcare costs. These findings also provide further evidence that policies encouraging prescribing of generic medicines could have substantial savings on healthcare expenditure and, if implemented in hospital formularies, could also influence prescribing outside of the hospital setting, resulting in further savings. However, in their analysis, the researchers assumed that the health outcomes of patients would be the same whatever type of drug they used (brand, generic, or follow-on), as they had no information on health outcomes. Nevertheless, this study provides useful information for healthcare providers and policy makers about the cost implications of the evergreening strategies used by the pharmaceutical industry, particularly for follow-on drugs.

**Additional Information:**

Please access these websites via the online version of this summary at http://dx.doi.org/10.1371/journal.pmed.1001460.

- This study is further discussed in a *PLOS Medicine* Perspective by Aaron Kesselheim
- Wikipedia provides an explanation of evergreening (note that Wikipedia is a free online encyclopedia that anyone can edit; available in several languages)
- The World Trade Organization has detailed information on TRIPS

## Introduction

To balance the at times competing goals of increasing access to new drugs on the one hand, and rewarding drug innovation via patents on the other hand, drug manufacturers are granted exclusive manufacturing rights for periods of up to 20 y [1]. This can generate large revenues that often exceed initial investments, thus providing an incentive for pharmaceutical companies to develop new drugs [2]. However, profits have increasingly come under pressure because of stricter regulatory procedures for drug approval, implementation of price control policies, and increased competition by generic drugs [3],[4]. Pharmaceutical companies have responded by developing a number of tactics to extend market monopoly. These are known as “evergreening” strategies, or more euphemistically as “life cycle management”, with sometimes questionable benefit to society [5].

One common strategy is the patenting and marketing of a single enantiomer of an already approved drug [6]. When large-scale production of enantiopure compounds was first possible in the 1980s, it was expected that the use of these drugs would translate into direct health benefits for the patient, e.g., in the form of better tolerability [6]. However, there is currently no clear evidence of increased efficacy or tolerability of enantiopure compounds over racemic combinations [5],[7]. The marketing of enantiopure compounds with questionable advantages over the original drug is just one example of an evergreening strategy [8]. Other evergreening techniques include patenting combination formulations, structural analogues, active metabolic types, and slow-release forms [9]. The specific impact of these second-generation products, or “follow-on” drugs, on overall healthcare costs has not been well studied.

Hospitals usually adopt a payer perspective strategy, trying to minimise acquisition costs for their medications [11]. Usually, pharmaceutical companies offer high rebates to hospitals on their brand or follow-on drugs to assure that the hospitals will buy and use their drugs, speculating that hospital prescription patterns may influence prescription patterns in the community in their favour [12]. The objective of our study was to assess the overall costs associated with the prescription of follow-on drugs as a result of an “evergreening” strategy over a 9-y period in the Swiss canton of Geneva. In addition, we aimed to calculate the financial impact of the Geneva University Hospitals (HUG) restrictive drug formulary (RDF) on overall healthcare costs, the so called “spillover effect” [10].

## Methods

### Study Population and Settings

The Swiss canton of Geneva has a single public hospital system (HUG) providing primary and tertiary care to a total population of 464,000 inhabitants (2010), with 2,000 beds (2008) and approximately 50,000 admissions and 800,000 outpatient visits each year. Community physicians account for an additional 1.2 million outpatient consultations per year [12].

The Swiss healthcare system provides mandatory health insurance with universal access to healthcare for everyone [13]. To encourage utilisation of generic medications, Swiss regulations have allowed pharmacists to substitute brand drug prescriptions with generic equivalents since 2001. In 2006, a 20% patient co-payment was introduced instead of the usual 10% for brand drug prescriptions, when brand drugs did not lower their price.

Like many other hospitals, HUG has implemented a RDF trying to minimise acquisition costs for medications (which may be well below the official market price for some drugs) and to limit the number of medications available in the hospital. Drugs are selected based on their efficacy, safety, and costs. For the purpose of this study we differentiated three settings: (1) inpatient setting: all the prescriptions generated during a hospitalisation, (2) hospital spillover setting: medications prescribed by HUG physicians but dispensed by community pharmacies (e.g., at hospital discharge or in outpatient clinics), and (3) community setting: drug prescriptions dispensed by community pharmacies and not issued by HUG physicians. These settings have different rules. Drug prices are negotiated and prescriptions are restricted for the inpatient setting, while in the other settings, prices are fixed and prescriptions are unrestricted.

### Follow-On Drugs Based on Evergreening Strategies

For each evergreening situation we differentiated three categories of drugs marketed at different prices: the initially patented drug, commonly called the brand drug (e.g., brand omeprazole); the generic version of the brand drug, marketed after patent expiration (e.g., generic omeprazole); and the follow-on drug, defined as the brand drug to which an evergreening strategy is applied (e.g., esomeprazole as an active isomer of omeprazole). The follow-on drug is usually marketed by the pharmaceutical company that owns the brand drug, and both drugs are marketed at the same time in most cases, effectively making them competitors.

We identified eight follow-on drugs available in the canton of Geneva between 1 January 2000 and 31 December 2008: three drugs for which an isomer had been marketed (levocetirizine as follow-on drug for cetirizine, escitalopram for citalopram, esomeprazole for omeprazole), one active metabolite (desloratadine for loratadine), two combination formulations of the originally patented drug (alendronic acid combined with colecalciferol for alendronic acid alone, simvastatin combined with ezetimibe for simvastatin alone), one slow-release formulation (zolpidem extended release), and one structural analogue (pregabalin for gabapentin).

To analyse the impact of evergreening strategies on overall healthcare spending, we calculated a monthly follow-on market share score—as an indicator of market competitiveness—as the percentage of follow-on drugs (in defined daily doses [DDDs]) of all prescriptions of follow-on, generic, and brand drugs in that category. Follow-on market share scores could therefore range from 0% (no use of the follow-on drug) to 100% (exclusive use of the follow-on drug) [14].

### Data Sources

We combined three different administrative registries for our analyses: the HUG hospital registry for patient characteristics, the HUG hospital pharmacy database for drugs dispensed in the inpatient setting, and the OFAC database for drugs dispensed both in the hospital spillover and the community settings. OFAC is a Swiss pharmacist professional organization that serves as an administrative intermediary for 92% of affiliated pharmacies and health insurance companies and covers 80% of the insured population in the canton of Geneva. The pharmacies not affiliated with OFAC are comparable with regard to patient population and location, but patients obtaining prescriptions at a non-affiliated pharmacy send their bills directly to their health insurer.

### Institutional Review Board Approval

The HUG Ethics Committee considered the study to be exempt from formal institutional review since it was based upon administrative data without direct patient involvement. All confidential health information was removed to create anonymous analytic datasets in conformity with Swiss data protection regulations.

### Costs Calculation and RDF Spillover Effect

“Extra costs” associated with brand and follow-on drug prescriptions in the community were calculated using the World Health Organization's recommended metric, the 2008 DDD, defined as the assumed average maintenance dose per day for a drug used for its main indication in an adult [15]. To analyse the impact of evergreening strategies on healthcare spending, we combined the hospital spillover and community settings. Costs were analysed under three scenarios assuming a replacement with the corresponding generic, when available, of (1) all brand drug prescriptions, (2) all follow-on drug prescriptions, and (3) both follow-on and brand drug prescriptions. The “extra cost” was assessed as the difference between the total cost based on the observed data and the total cost estimated in the three scenarios. Costs were converted from Swiss francs to Euros at the established 2011 exchange rate of €1 = 1.20 CHF. Inflation was not taken into account.

### RDF Spillover Effect

We defined the RDF spillover effect by comparing the follow-on market share for the three settings outlined above, i.e., inpatient, hospital spillover, and community. We hypothesised that hospital physicians accustomed to prescribing according to the RDF for their inpatients would be influenced when prescribing in the outpatient clinic, the emergency department, and at discharge, even if under these conditions prescriptions are unrestricted. We analysed the monthly dynamic RDF spillover effects for the only two evergreening strategies that were directly affected by a change in the HUG RDF during the study period. At admission, all patients using a proton-pump inhibitor were switched to the follow-on drug esomeprazole from 1 October 2002 onwards, and all patients on cetirizine and levocetirizine were switched to generic cetirizine from 1 December 2004 onwards.

### RDF-Spillover-Associated Costs

To explore the spillover costs or benefits we hypothesised that if the hospital would not have implemented a RDF, the follow-on market share of the hospital spillover and community settings would be equivalent and thus represent the market-driven force. We defined the financial spillover-associated costs as the difference in market share between these two settings. We therefore applied the community follow-on market share to that of the hospital spillover setting and calculated the corresponding “extra costs”.

### Statistical Analysis

Demographic variables were expressed as percentages or means with standard deviation. The global “extra costs” and those related to the spillover were assessed using a simulation-based approach. First, monthly community drug consumption was simulated (for each drug and reference) to reproduce the observed data and to introduce the variability of monthly drug consumption into the cost calculation. Monthly drug consumption values were generated from a normal distribution with mean and variance derived from the observed data. A one-way sensitivity analysis was conducted by varying the correlation between successive months from 0.0 to 0.5, and a correlation of 0.5 was selected in a conservative way (95% confidence interval was larger). The changes in consumption over time were captured by this simulation procedure. These simulated data corresponded to the base-case scenario. We checked graphically whether the generated monthly drug consumption values fitted to the effective medication use. Second, drug consumption was extrapolated under the three scenarios (generic replacement of brand drugs, follow-on drugs, or both) by applying new prescription rates to the simulated data. In the first scenario, the prescription rate of brand drugs was set to 0 if a generic was available, and the brand drug DDDs were transferred to generic equivalence. The model uncertainty related to the extrapolations was accounted for by introducing a random effect on the prescription rates of generic references: the DDDs transferred to generic drugs were split in the references with rates that varied by 30% (relatively) compared to the observed data. The “extra costs” were the cost differences between the base-case scenario and each of the three scenarios, and the simulations were run 10,000 times. The reported results were the mean extra costs and the 95% confidence interval (percentiles 0.025 and 0.975 of the set of 10,000 values of extra costs). We used a similar approach to derive extra costs from the spillover. Details are given in Text S1. Simulations were performed with R 2.15.1 software (R Foundation for Statistical Computing).

The spillover effect dynamic was analysed under robust time series analysis using autoregressive integrated moving average models according to the Box-Jenkins methodology, which allows the stochastic dependence of consecutive data to be modelled [16],[17]. We used dummy variables (0 before intervention, 1 after) to assess changes in level and slope after introduction of a RDF in the hospital and generics coming to market. Significance tests for parameter estimates at a *p*-value of <0.05 were used to eliminate the unnecessary terms. Among different models, we chose the most parsimonious one, i.e., the model with the fewest parameters. All final model residuals passed a “white noise” test (based on Ljung-Box statistics). R2 represents the overall fitting of a model. Statistical analysis was performed with Eviews 7 software (QMS).

## Results

### Study Population Characteristics

During the study period the number of patients receiving either a brand or follow-on product increased from 56,686 patients in 2001 to 131,193 patients in 2008. The most commonly prescribed follow-on medications were esomeprazole and escitalopram (55% and 32% of all patients prescribed follow-on drugs over the entire study period, respectively). Table 1 summarises study population characteristics and medications prescribed.

**Table 1:** Characteristics of patients and medication prescriptions in the hospital and community.

Characteristic	Year							
	2001 (*n* = 56,686)	2002 (*n* = 72,778)	2003 (*n* = 82,269)	2004 (*n* = 94,920)	2005 (*n* = 103,455)	2006 (*n* = 118,483)	2007 (*n* = 130,070)	2008 (*n* = 131,193)
**Gender, ** ***n*** ** (percent)** a								
Male	24,861 (44)	28,488 (39)	32,436 (39)	37,276 (39)	39,685 (38)	46,494 (39)	51,683 (40)	52,864 (40)
Female	31,825 (56)	44,290 (61)	49,833 (61)	57,644 (61)	63,770 (62)	71,989 (61)	78,387 (60)	78,329 (60)
**Mean age** a **(± standard deviation)**	52.0 (27.0)	52.19 (20.0)	53.3 (20.1)	54.8 (21.4)	54.8 (21.3)	55.1 (21.3)	55.1 (21.3)	56.3 (21.2)
**Number of patients receiving brand and follow-on drugs** b **(hospital and community)**								
Alendronic acid (brand)	1,789	2,824	4,009	5,064	5,727	6,140	5,548	4,373
Alendronic acid and colecalciferol (follow-on)	—	—	—	—	—	594	1,186	1,019
Cetirizine (brand)	11,449	10,900	12,138	9,633	7,051	8,814	9,319	8,723
Levocetirizine (follow-on)	—	—	—	2,977	5,966	6,188	6,922	6,201
Citalopram (brand)	8,472	8,705	8,193	8,487	8,116	7,871	7,638	7,189
Escitalopram (follow-on)	—	1,335	3,762	4,888	5,125	5,985	6,714	7,103
Loratadine (brand)	5,305	6,247	4,772	4,953	4,515	3,743	3,520	3,428
Desloratadine (follow-on)	290	3,744	5,049	6,158	5,892	6,646	7,188	6,729
Gabapentin (brand)	617	1,107	1,656	2,322	2,544	2,411	2,422	2,758
Pregabalin (follow-on)	—	—	—	—	2,500	6,154	8,558	13,739
Omeprazole (brand)	15,450	15,839	15,394	18,889	22,872	25,083	27,797	27,064
Esomeprazole (follow-on)	1,200	3,968	6,944	10,557	13,894	18,845	22,765	27,385
Simvastatin (brand)	5,220	5,787	6,615	6,610	7,330	8,662	8,663	8,931
Simvastatin and ezetimibe (follow-on)	—	—	—	—	—	1,204	2,068	2,371
Zolpidem (brand)	9,144	10,598	11,770	12,854	13,971	14,533	16,269	16,350
Zolpidem extended release (follow-on)	—	—	—	—	—	1,163	2,470	2,353

a Each patient is counted once, even if several medications from the list are used.

b Patients count several times, if several medications from the list are used.

### Costs and “Extra Costs” Associated with Brand and Follow-On Drug Prescriptions in the Community

**Figure 1:**
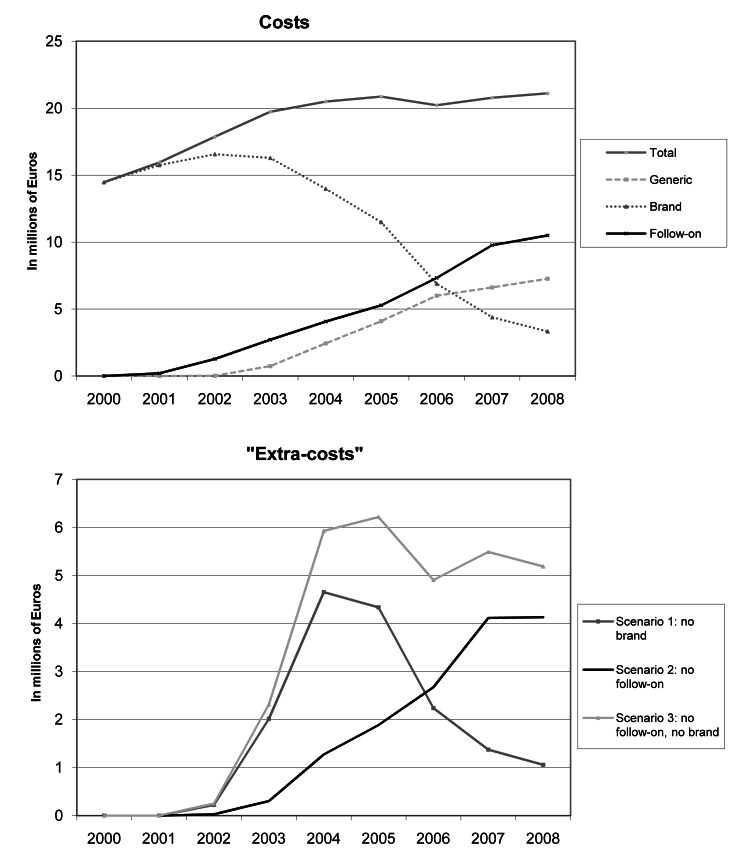
Costs and “extra costs” of brand, follow-on, generic, and total prescriptions in millions of Euros.

Figure 1 demonstrates that between 2000 and 2008, the total cost for all studied drugs was €171.5 (95% CI 170.2; 172.9) million. By category of drug, the total cost was €103.2 (95% CI 102.0; 104.3) million for brand drugs, €41.1 (95% CI 40.6; 42.0) million for follow-on drugs, and €27.2 (95% CI 26.8; 27.6) million for generics. Based on the “extra costs” calculated from scenario 1 (generic replacement of brand drugs) and scenario 2 (generic replacement of follow-on drugs), the healthcare system could have saved, over the entire study period, €15.9 (95% CI 15.5; 16.2) million and €14.4 (95% CI 14.1; 14.7) million if brand and follow-on drug prescriptions, respectively, had been replaced. This amounts to €30.3 (95% CI 29.8; 30.8) million over the entire study period if both brand and follow-on drug prescriptions were replaced at their corresponding community generic selling price equivalents when available (scenario 3).

It is noteworthy that “extra costs” attributable to brand drug prescriptions increased sharply between 2002 until 2004. This is a consequence of the increasing availability of generic counterparts: citalopram from 1 October 2002, omeprazole from 1 July 2003, simvastatin from 1 May 2004, cetirizine from 1 September 2004, zolpidem from 1 August 2005, loratadine from 1 July 2006, and alendronic acid and gabapentin from 1 July 2007. After 2004, the potential savings of replacing brand-name drugs with generics gradually decreased, particularly from 2006 onwards, when generic substitution through an additional co-payment cost containment policy was incentivised in Switzerland (Figure 1). This decrease was fully offset by the progressive increase in costs due to the replacement of brand drugs by follow-on prescriptions in the community. Table 2 illustrates the impact of each follow-on drug on total “extra costs” over the study period by scenario. Esomeprazole (41.5%), escitalopram (31.7%), and the combination of simvastatin and ezetimibe (17.6%) were the major contributors to these “extra costs”.

**Table 2:** “Extra costs” in millions of Euros, 95% CIs, and percent of total prescriptions for three scenarios.

Brand—Follow-On Drug	Scenario 1 (Brand Drugs Replaced with Generic)			Scenario 2 (Follow-On Drugs Replaced with Generic)			Scenario 3 (Brand and Follow-On Drugs Replaced with Generic)		
	Euros (Millions)	95% CI	Percent of Total	Euros Millions)	95% CI	Percent of Total	Euros (Millions)	95% CI	Percent of Total
Omeprazole—esomeprazole	7.4	7.2; 7.6	46.3%	5.2	5.0; 5.4	36.2%	12.6	12.3; 12.9	41.5%
Citalopram—escitalopram	4.8	4.6; 5.0	30.0%	4.8	4.7; 5.0	33.6%	9.602	9.3; 9.9	31.7%
Simvastatin—simvastatin and ezetimibe	2.7	2.5; 2.9	17.0%	2.6	2.5; 2.8	18.3%	5.337	5.1; 5.6	17.6%
Alendronic acid—alendronic acid and colecalciferol	0.2	0.2; 0.2	1.3%	0.2	0.2; 0.22	1.4%	0.406	0.4; 0.4	1.3%
Zolpidem—zolpidem extended release	0.4	0.4; 0.5	2.6%	0.1	0.1; 0.1	0.4%	0.482	0.5; 0.5	1.6%
Loratadine—desloratadine	0.1	0.1; 0.1	0.3%	0.3	0.2; 0.4	2.2%	0.354	0.3; 0.4	1.2%
Gabapentin—pregabalin	0.2	0.1; 0.2	1.0%	0.7	0.6; 0.7	4.7%	0.823	0.8; 0.9	2.7%
Cetirizine—levocetirizine	0.2	0.2; 0.3	1.5%	0.5	0.4; 0.5	3.3%	0.710	0.7; 0.8	2.3%
Total	15.9	15.5; 16.2		14.4	14.1; 14.7		30.3	29.8; 30.8	

### Spillover Effect and Associated “Extra Costs”

#### Esomeprazole spillover

**Figure 2:**
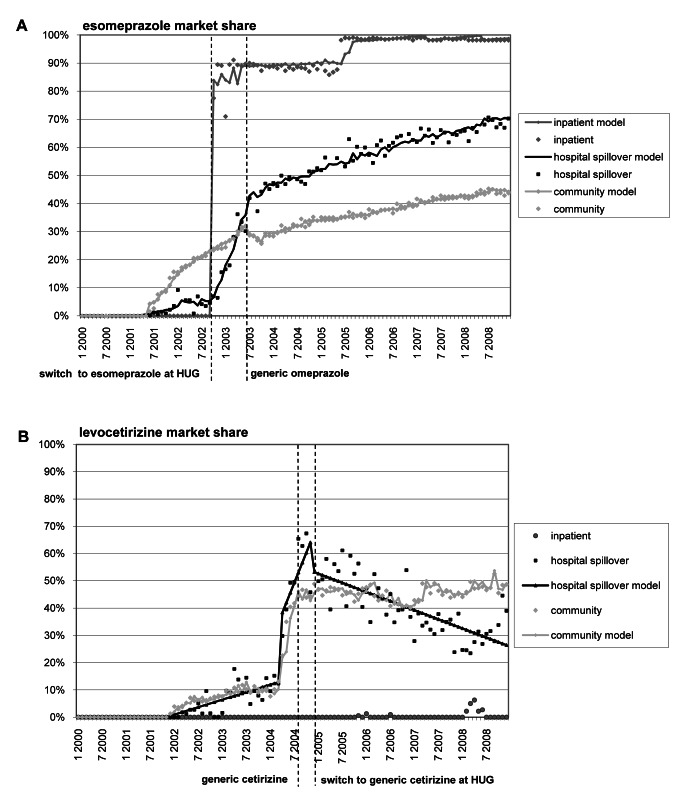
Esomepraprole and levocetirizine market share. This figure shows changes in esomepraprole (A) and levocetirizine (B) market share before and after changes for these drugs in the HUG RDF and generics coming to market.

The RDF spillover effect is illustrated for esomeprazole (Figure 2A) and generic cetirizine (Figure 2B) in Figure 2. From October 2002, when the HUG RDF switched to esomeprazole, until July 2003, when generic omeprazole was marketed, the esomeprazole hospital spillover market share moved from 5.2% (*p*<0.05) to 35.8% (*p*<0.05). During this same period, no statistically significant change in trend or level was observed in the community setting. From July 2003 onwards, we observed a statistically significant increase in trend in both hospital spillover and community settings, leading to 70.3% (*p*<0.05) and 41.0% (*p*<0.05) esomeprazole market share, respectively, in December 2008. We found a significant first-order correlation (*p*<0.05) for the hospital spillover setting, and R2 for both autoregressive integrated moving average models was 99%.

#### Generic cetirizine spillover

Six months prior to the generic drug entering the market (September 2004), the pharmaceutical company producing brand cetirizine removed the drug from the reimbursement list, shifting the levocetirizine market share from 12.8% (*p*<0.05) to 56.7% (*p*<0.05) in the hospital spillover setting, and from 10.2% (*p*<0.05) to 43.2% (*p*<0.05) in the community setting. From December 2004 onwards, when the RDF switched from brand to generic cetirizine, we observed a statistically significant decrease in trend only in the hospital discharge setting, leading to a 26.4% (*p*<0.05) levocetirizine market share in December 2008, compared to 48.6% (*p*<0.05) in the community setting. There was no autocorrelation for the hospital spillover setting. R2 was 90% for the hospital spillover setting and 98% for the community setting.

#### Spillover-associated extra costs

Table 3 shows the time frame of the specific decisions taken by the hospital for its RDF and the associated “extra costs” of these decisions. It demonstrates that the diffusion of hospital prescription patterns into the community resulted in an “extra cost” of €503,600 (95% CI 444,500; 563,100) over the study period, mainly attributable to esomeprazole (€330,300 [95% CI 276,100; 383,800]) and escitalopram (€192,300 [95% CI 168,500; 215,900]). However, we demonstrated that spillover can also be beneficial for the healthcare system, e.g., if a generic drug is listed in the RDF (cetirizine −€7,700 [95% CI −11,100; −4,100]).

**Table 3:** Time frame of changes in the hospital drug formulary (RDF) and spillover-associated “extra costs” (95% CI) in thousands of Euros.

Brand—Follow-On Drug	Changes in the RDF	Spillover-Associated Extra Costs, in Thousands of Euros (95% CI), for Each Year								
		2001	2002	2003	2004	2005	2006	2007	2008	2000–2008
Omeprazole—esomeprazole	All the proton-pump inhibitor prescriptions are switched to esomeprazole at admission from 1 October 2002	13.2 (8.4; 18.9)	52.1 (40.7; 63.9)	0.2 (−19.4; 19.6)	24.6 (1.3; 48.1)	32.1 (5.5; 57.5)	68.3 (45.5; 90.7)	84.5 (64.2; 104.8)	55.2 (43.9; 66.7)	330.3 (276.1; 383.8)
Citalopram—escitalopram	RDF switched to generic citalopram from 1 December 2003; escitalopram is unrestricted	0^a^	1.7 (0.7; 2.9)	6.3 (4.6; 8.2)	26.2 (19.4; 33.4)	37.0 (27.2; 47.3)	51.2 (37.9; 64.8)	40.0 (28.0; 52.4)	29.8 (21.4; 38.0)	192.3 (168.5; 215.9)
Gabapentin—pregabalin	RDF switched to generic gabapentin from 1 February 2008; pregabalin is unrestricted	0^a^	0^a^	0^a^	0^a^	0.9 (−0.2; 1.9)	1.4 (−1.2; 4.1)	1.4 (−0.3; 3.3)	1.3 (−0.4; 3.1)	5.0 (1.3; 8.8)
Zolpidem—zolpidem extended release	RDF switched from brand zolpidem to generic from 1 June 2006; extended-release zolpidem is restricted	0^a^	0^a^	0^a^	0^a^	0.7 (0.3; 1.1)	1.6 (0.6; 2.5)	1.1 (0.5; 1.6)	1.0 (0.5; 1.5)	4.3 (3.0; 5.6)
Loratadine—desloratadine	RDF switched from brand to generic loratadine, and from desloratadine to generic cetirizine at admission from 1 February 2006	0.0 (−0.2; 0.0)	0.0 (0.0; 0.2)	0.0 (−0.1; 0.1)	0.0 (−0.1; 0.2)	0.0 (−0.1; 0.2)	0.6 (−0.1; 1.8)	1.0 (0.0; 2.6)	0.7 (0.0; 1.6)	2.4 (0.7; 4.5)
Alendronic acid—alendronic acid and colecalciferol	Neither alendronic acid nor its combination is included in the RDF; prescriptions are unrestricted	0^a^	0^a^	0^a^	0^a^	0^a^	0.0 (−0.1; 0.0)	−0.8 (−2.1; 0.5)	0.1 (−0.9; 1.1)	−0.7 (−2.4; 1.0)
Cetirizine—levocetirizine	RDF switched from brand cetirizine and follow-on levocetirizine to generic at admission from 1 December 2004	0.1 (0.0; 1.2)	0.6 (0.2; 2.5)	0.0 (0.0; 0.0)	0.2 (−0.1; 0.7)	0.0 (−1.3; 1.5)	−0.4 (−2.4; 1.5)	−8.2 (−10.3; −6.1)	0.0 (−0.1; 0.1)	−7.7 (−11.1; −4.1)
Simvastatin—simvastatin and ezetimibe	RDF switched from brand simvastatin to generic from 1 August 2004; combined simvastatin and ezetimibe is unrestricted	0^a^	0^a^	0^a^	8.4 (3.0; 14.2)	22.0 (15.8; 28.1)	−10.7 (−15.0; −6.7)	−22.5 (−29.4; −16.0)	−19.6 (−27.8; −11.7)	−22.3 (−36.7; −8.2)
Total		13.3 (8.5; 19.0)	54.5 (43.0; 66.1)	6.5 (−13.1; 26.0)	59.5 (34.3; 84.6)	92.7 (63.7; 121.2)	112.0 (85.4; 139.1)	96.7 (71.9; 121.6)	68.5 (52.2; 84.9)	503.6 (444.5; 563.1)

a No generic available.

## Discussion

Our study demonstrates that the evergreening strategies of the pharmaceutical industry have been successful in the canton of Geneva with regard to several brand drugs facing intense price competition from generics after losing their patent protection. The generic competition and co-payment incentive implemented in Switzerland in 2006 most likely contributed to an increasing replacement of brand with generic drugs and also reduced prices for brand drugs [18]. However, we found that this effect was fully offset by the successful marketing of follow-on drugs.

We demonstrate that the total healthcare expenditure “volume” for the examined drugs was constant over the study period, despite the increasing availability of cheap generics; this is comparable to the “squeezing the balloon” phenomenon [19]. These results suggest that, absent other policies and strategies to offset evergreening marketing tactics, the potential of generic medicines as a key strategy to decrease drug costs is unlikely to be successful.

While the patent system's main purpose is to incentivise innovation, it is sometimes used (or abused) to stifle competition. A recently published study identified 108 patents for two antiretroviral drugs (ritonavir and lopinavir/ritonavir) that could delay competition with generics until at least 2028, well beyond the usual 20-y period [9]. Some of these patents were judged by Amin et al. [9] to shelter “innovations” of very limited value. A similar argument may be made for some of the follow-on drugs examined in this study. The results of several studies suggest that the clinical benefit of follow-on drugs over the original brand drugs (or their generics) is unclear or marginal at best [5],[6],[20],[21]. To determine the true benefits of follow-on drugs, well-conducted randomised controlled clinical trials comparing them with the corresponding brand-name or generic drugs at equivalent dosages would be necessary, but few of these trials exist, and for most follow-on drugs, it is unlikely that these trials will ever be conducted [21],[22]. In order to minimise the impact of follow-on drug prescriptions on healthcare costs in the United Kingdom, Hughes and colleagues have suggested that before “evergreened” drugs are marketed, they should first undergo a cost-efficacy comparison process with their generic or brand-name counterparts [5],[23]. In the absence of direct comparative data, however, this may prove difficult.

While cost containment policies seem vital in a time of steadily increasing healthcare costs in many Western countries, it should be kept in mind that excessive pressure on pharmaceutical companies may also lead to the unintended consequence of reduced investment in innovation. Therefore, Hitchings et al. recently recommended that policy makers not cut follow-on drug access, but alert prescribers and medical students about evergreening strategies [7]. It remains to be seen if such an approach can be successful, given that it would have to compete with the intense promotional activities by pharmaceutical representatives in many settings [24].

Our study also confirmed the “spillover effect” of the hospital RDF on prescriptions in the community, leading to an increase in healthcare expenditures as a whole. Other studies have found similar results. Feely et al. found that hospital-initiated prescriptions were responsible for an increase in the volume and subsequent cost of prescriptions in general practices in Ireland [25]. Another study in California demonstrated that physicians who had many patients receiving Medicaid (the program for low-income families and individuals) generated a significant increase in prescriptions of drugs on Medicaid's drug list in their non-Medicaid-affiliated patients [10].

To our knowledge, our study is the first to show the specific impact on costs of follow-on drugs integrated into a hospital RDF. We demonstrated that this can influence prescription patterns in the community and benefit drug manufacturers: gains generated by increased prescription of follow-on drugs in the community through the “spillover phenomenon” can greatly exceed the cost of rebates offered to hospitals. On the other hand, we also showed with the example of generic cetirizine that a RDF can contribute to reduced overall costs for the healthcare system. Furthermore, our study illustrates that the drug manufacturer's removing brand cetirizine from the reimbursement list prior to the generic drug coming onto the market accelerated the therapeutic switch from brand cetirizine to levocetirizine both in the hospital spillover and community settings.

### Strengths and Limitations

This study has several strengths. First, we used a single data source to analyse the financial impact of follow-on and brand prescriptions in the canton of Geneva over a 9-y period. This database includes more than 73% of the total of insured patients, thus guaranteeing a uniform and large data collection system [26]. Second, we analysed a specific geographical area that includes not only one major public, university-affiliated hospital but also private clinics and physician practices. This made it possible to measure the interaction between hospital and community prescriptions and to show how the hospital contributed to increased healthcare costs by taking an exclusive payer perspective when selecting follow-on drugs into its RDF. Third, by measuring prescriptions over an 9-y time period we not only were able to measure the follow-on market share at a given time point, but—using time series analysis—could also demonstrate that a hospital RDF can have a significant impact on drug prescriptions.

Our study also has several limitations. First, we assumed that health outcomes for patients would be the same regardless of which type of drug was prescribed (brand, generic, or follow-on). Whether this assumption is correct in all cases still needs to be demonstrated [5],[27]. Second, the three scenarios analysed were based on the assumption that all brand and/or follow-on prescriptions would be switched to generics. This approach does not take into account the fact that some patients may prefer the galenic formulation of certain brand or follow-on drugs and may thus be reluctant to switch to the generic equivalent [28]. Third, we were also unable to measure the impact on adherence to treatment and health outcome of the substitution of patients' personal medications for RDF drugs at hospital admission [29]. Fourth, our study analysed only a single Swiss canton, limiting the generalisability of our findings. Finally, we did not examine complementary strategies developed by drug manufacturers to promote brand and follow-on drugs, such as physician education and visits of pharmaceutical representatives.

### Conclusion

Drug manufacturers have developed various “evergreening” strategies that contribute to increased overall healthcare costs. The study provides further evidence that cost-saving policies encouraging generic medicine prescriptions, which can have substantial savings for healthcare expenditures, may be offset by increased costs from follow-on drugs. A hospital's attempts to minimise its own medication costs can, as an unintended consequence, lead to increased overall community healthcare expenditure through “spillover effects”.

## Supporting Information

Text S1 **Statistical method for the estimation of the costs, “extra costs” associated with brand and follow-on drug prescriptions in the community, and spillover “extra costs”.**
(DOCX)

## Abbreviations

DDD: defined daily dose
HUG: Geneva University Hospitals
RDF: restrictive drug formulary
