# Supplementary material for: Patented Drug Extension Strategies on Healthcare Spending: A Cost-Evaluation Analysis
Source: PLoS Med. 2013 Jun 4;10(6):e1001460. doi: 10.1371/journal.pmed.1001460 (PMC3672218; doi:10.1371/journal.pmed.1001460)
Supplement: Text S1 — Statistical method for the estimation of the costs, “extra costs” associated with brand and follow-on drug prescriptions in the community, and spillover “extra costs”. (DOCX) [file pmed.1001460.s001.docx]

Supplement: Statistical method for the estimation of the costs, “extra-costs” **associated with brand and follow-on drug prescriptions in the community** and spillover “extra-costs”.

**Cost and extra-costs associated with brand and follow-on drug prescriptions in the community**

In the baseline scenario (corresponding to the observed data in the database from OFAC), we generated 10’000 simulated sets of data of monthly drug consumption in community. Thus, for each simulation, we extrapolated the drug consumption with the three scenarios:

1)   Daily define doses (DDD) of follow-on were replaced by DDD of generic (when generic was available)

2)   Daily define doses (DDD) of brand were replaced by DDD of generic (when generic was available)

3)   Daily define doses (DDD) of follow-on and brand replaced by DDD of generic (when generic was available)

The “extra-cost” for each scenario was the difference between each scenario and the baseline scenario. The purpose of the simulation scheme is to capture the variability of the monthly drug consumptions and to transfer it in the computation of the “extra-costs”. Moreover, uncertainty related to the extrapolation is introduced the simulated data in the scenario 1, 2 and 3. We detail the statistical method in three steps: 1) the generation of the data for the baseline scenario (according to the observed data OFAC); 2) the calculation of the cost in the baseline scenario; 3) the extrapolation of the data for scenario 1 (the procedure is similar for the other scenarios).

Step 1): The data of monthly drug consumption in the baseline scenario were simulated as follows:

-    A lowess curve (using locally-weighted polynomial regression) was computed to assess the mean of monthly DDDs over time. The function “lowess” of the R package *stats* was used. Several smoother pans were explored and we selected a smoother pan providing an acceptable goodness-of-fit. This lowess curve captured the evolution of the consumption. An illustration is given in Figure 1A for one the most important reference of omeprazole.

-    The variability of the monthly DDDs was assessed by the standard deviation of the difference between the mean DDDs and the observed values. This calculation was made yearly to allow the variability to change over time. In Figure 1B, we represented the differences between the lowess curve and the observed data.  A normal distribution to model this difference was compatible with the observed data.

-    The monthly DDDs were generated using a normal distribution. The mean of the distribution for each month was the value of the lowess curve. The variance was derived from the difference between the lowess and the observed data (previous step). Moreover, we introduced a correlation coefficient between two successive DDDs. Thus, the DDDs were randomly generated from a multivariate normal distribution (using the function rmvnorm of the R package *mvtnorm*). We ranged the correlation coefficient between 0.0 and 0.5, and the confidence intervals of the results increased with the correlation. To be conservative, we present the results for a correlation of 0.50. An example of simulated data is represented in Figure 1D.

-    The generation of monthly DDDs was performed for each reference of generic, brand and follow-on drugs, and for each drug (Omeprazole, Cetirizin, etc).

-    10’000 set of data were simulated

Step 2): From the simulated data in the baseline scenario (Step 1), the mean cost and the 95% interval as follow were calculated as follow:

-    The cost related to each reference was calculated by the sum over months of the monthly DDDs multiplied by the tariff of the reference

-    The cost of generic (respectively brand, follow-on) was the sum of the cost over all the references of generic (brand, follow-on)

-    The total cost was the sum of the costs of generic, brand and follow-on

-    As 10’000 set of data were simulated, the mean cost was the average on the 10’000 values of cost. The 95% confidence interval was obtained by the percentiles 0.025 and 0.975. The distribution of costs were represented graphically (histogram) and were found approximately gaussian.

Step 3): The next step consisted in the extrapolation of the data for the scenarios 1, 2 and 3. The process is similar for the 3 scenarios and, for convenience; we described the process only for the scenario 1, in which the DDDs of brand are assumed to be replaced by DDDs of generic.

-    When at least one reference of generic was available, the DDDs of brand were assigned to references of generic. This step was performed each month.

-    The distribution of the replaced DDDs of brand among the references of generic was based on the observed distribution of generic. For instance, if a given reference of generic corresponded to 10% of the generic consumption, around 10% of the replaced DDDs of brand were affected to this reference of generic. However, a random effect was introduced in this distribution to capture the uncertainty in the relocation of the DDDs of brand:

o   for each reference of generic, a random value was generated from a uniform distribution U[-0.25;0.25] and its exponential was taken.

o   The distribution of each reference of generic was multiplied by the obtained value in the previous step

o   The distribution of all references of generic was rescaled for the sum to equal 1

o   For instance, for a reference corresponding to 10% of the consumption of generics, if the random number is -0.2, then 0.10*exp(-0.2)=0.08 (ie 8%) of the DDDs of brand will be replaced by this reference of generic. After rescaling, the distributions of references of generic could vary (relatively) by +/-30%.

-    The extrapolated data in the scenario 1 were obtained by:

o   removing the DDDs of brand from the simulated data of the baseline scenario

o   adding to the DDDs of generic, the DDDs of brand distributed in the different references of generic according to distribution previously computed.

-    The previous step of relocation of DDDs of brand was applied on each of the 10’000 set of data simulated fir the baseline scenario. The mean cost and its 95% confidence interval were obtained by similar calculations than for the baseline scenario.

-    For each iteration, the difference between the baseline scenario and the scenario 1 (“extra-cost”) was calculated and a set of 10’000 values of “extra-costs” was obtained. The mean “extra-cost” was the mean over the 10’000 values and the 95% confidence interval was obtained by the percentiles 0.025 and 0.975.

The goodness-of-fit of the simulation method was checked by comparing graphically the observed and the simulated monthly DDDs of generics, brands and follow-on. An example is represented in Figure 2.

Figure 1 of the supplement: Simulation of monthly drug consumptions in community. The example of the reference of Omeprazole.(ANTRA MUPS, 2054158, code ATC: A02BC01)

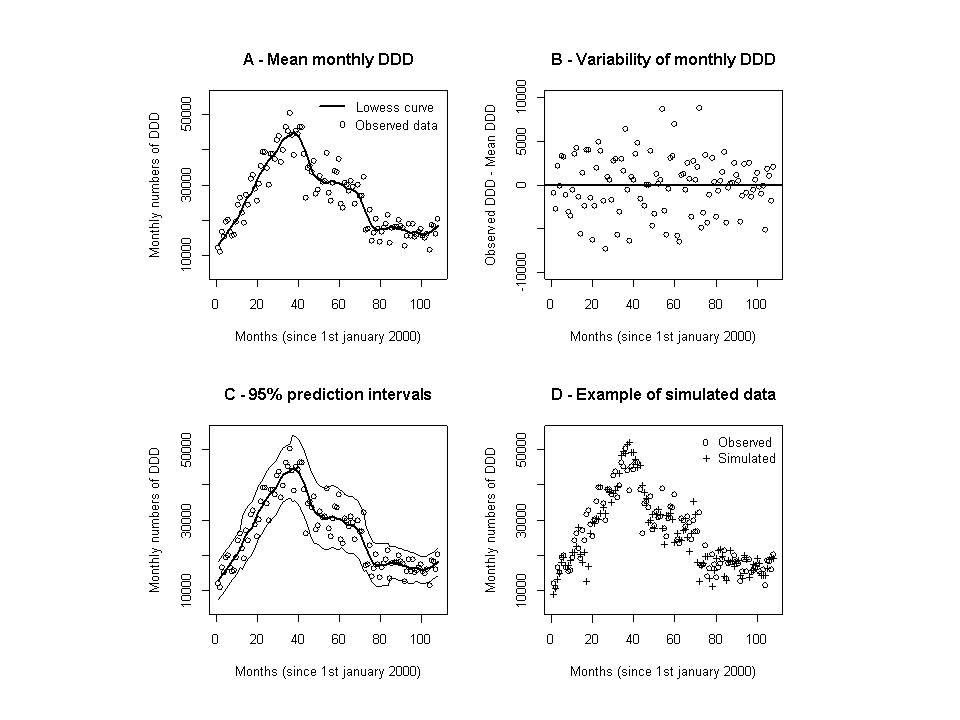

Figure 2 of the supplement: Representation of the observed and the simulated monthly drug consumption of Omeprazole. N=100 iterations are represented. This representation allows checking the goodness-of-fit of the simulation scheme.


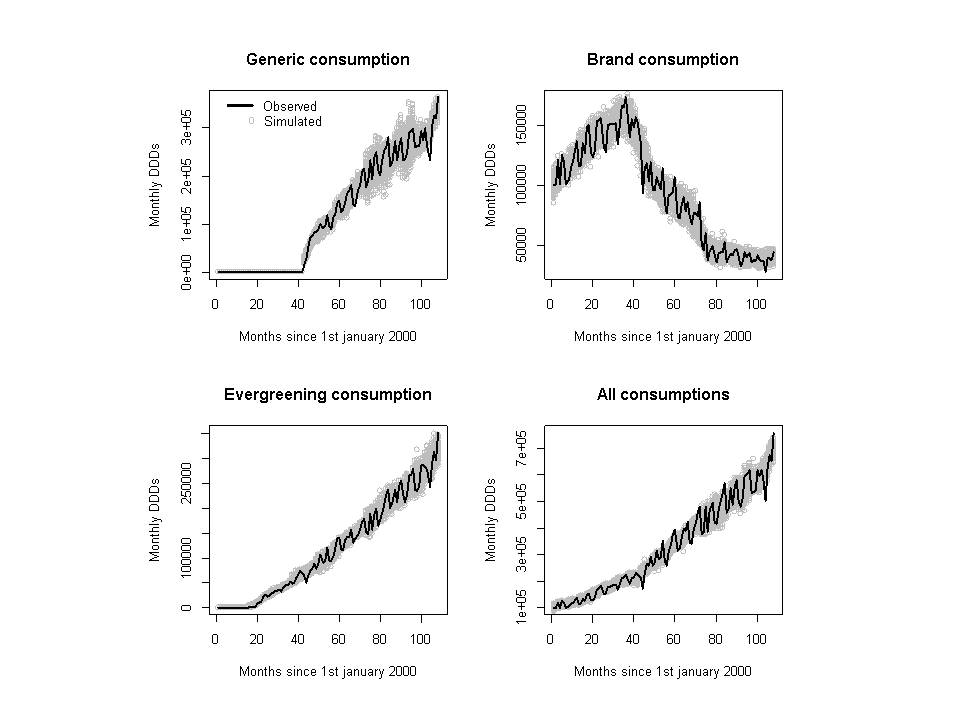


**Spillover “extra-costs”**

The spillover “extra-costs” is defined as the difference between the cost of drug consumption for the hospital spillover setting and the expected cost under the assumption that the rates of generic, follow-on and brand prescriptions in community are applied to the prescriptions at the hospital spillover setting. We used a simulation based approach to assess the spillover effect. A total of 10’000 iterations were performed in the simulation scheme. At each iteration, we generated a set of monthly drug consumptions for the hospital spillover setting. For this step, we used the OFAC database. The purpose of this step was to estimate the proportion of DDDs prescribed for generic, brand and follow-on at the hospital spillover setting. The same analysis was performed on the data of drug consumption in community (OFAC database), and the rates of generic, brand and follow-on prescriptions in community were assessed. These rates were extrapolated to the prescriptions at the hospital spillover setting. Finally, the cost of drug consumptions was calculated for the drug consumption at hospital spillover setting and for the extrapolated consumption.

For each of iteration of the simulation (N=10’000 iterations), we followed 5 steps in the simulation scheme:

-    Step 1): Generation of monthly drug consumption at the hospital spillover setting from OFAC. The approach for generating these data is similar to the step 1) for the “extra-costs”.

o    the monthly number of generic, brand and follow-on were computed from OFAC database. This corresponds to the observed data

o    a lowess curve (using locally-weighted polynomial regression) was computed to assess the mean of monthly DDDs over time

o    the variability of the monthly DDDs was assessed by the standard deviation of the difference between the mean DDDs and the observed values. This calculation was made yearly to allow the variability to change over time.

o    the monthly DDDs were generated using a multinormal distribution. The mean of the distribution for each month was the value of the lowess curve. The variance was derived from the difference between the lowess and the observed data. A correlation coefficient of 0.5 between two successive DDDs was introduced. Thus, the DDDs were randomly generated from a multivariate normal distribution.

-    Step 2): Determination of prescription rates of generic, brand and follow-on in community. The approach is similar than in the Step 1). The OFAC database was used.

-    Step 3): The prescription rates of generic, brand and follow-on in community were extrapolated to the simulated data of patients at the hospital spillover setting:

o   a random effect was introduced in the prescription rate in community. For that, we used the approach described in the Step 3) of “extra-costs”

o   the total number of DDDs at the hospital spillover setting was multiplied by the prescription rates in the community setting, providing the extrapolated numbers of DDDs of generic, follow-on and brand at the hospital spillover setting.

-    Step 4): For generic (respectively brand and follow-on), the extrapolated number of DDDs at the hospital spillover setting was distributed into the corresponding references according to the rates calculated in the observed data. We also introduced a random effect in these rates (similarly to the Step 3 of “extra-costs”).

-    Step 5): the cost of drug consumption at the hospital spillover setting was computed for both simulated and extrapolated data by multiplying the DDDs of each reference by the corresponding tariff at hospital.

This simulation procedure was applied to each of the drugs (Omeprazole, Simvastin, etc) and the spillover effect was obtained by subtracting the cost computed on the extrapolated data to the cost computed on the data in simulated the Step 1. The reported results were the spillover averaged on the 10’000 iterations and the 95% confidence interval (percentiles 0.025 and 0.975).
